# Optimizing age-related hearing risk predictions: an advanced machine learning integration with HHIE-S

**DOI:** 10.1186/s13040-023-00351-z

**Published:** 2023-12-14

**Authors:** Tzong-Hann Yang, Yu-Fu Chen, Yen-Fu Cheng, Jue-Ni Huang, Chuan-Song Wu, Yuan-Chia Chu

**Affiliations:** 1 Department of Otorhinolaryngology, Taipei City Hospital, Taipei, 100 Taiwan; 2 General Education Center, University of Taipei, Taipei, 10671 Taiwan; 3 Department of Speech-Language Pathology and Audiology, National Taipei University of Nursing and Health Sciences, Taipei, 112303 Taiwan; 4 Department of Otolaryngology-Head and Neck Surgery, School of Medicine, National Yang Ming Chiao Tung University, Taipei, Taiwan; 5 Department of Medical Research, Taipei Veterans General Hospital, Taipei, 112 Taiwan; 6 School of Medicine, National Yang Ming Chiao Tung University, Taipei, 112 Taiwan; 7 Department of Otolaryngology-Head and Neck Surgery, Taipei Veterans General Hospital, Taipei, 112 Taiwan; 8 Institute of Brain Science, National Yang Ming Chiao Tung University, Taipei, 112 Taiwan; 9 College of Science and Engineering, Fu Jen University, Taipei, 243 Taiwan; 10 Information Management Office, Taipei Veterans General Hospital, Taipei, 112 Taiwan; 11 Big Data Center, Taipei Veterans General Hospital, Taipei, 112 Taiwan; 12 Department of Information Management, National Taipei University of Nursing and Health Sciences, Taipei, 112 Taiwan

**Keywords:** Age-related, Hearing loss, LGBM, Machine learning, HHIE-S, Predictive enhancement, Innovation

## Abstract

**Objectives:**

The elderly are disproportionately affected by age-related hearing loss (ARHL). Despite being a well-known tool for ARHL evaluation, the Hearing Handicap Inventory for the Elderly Screening version (HHIE-S) has only traditionally been used for direct screening using self-reported outcomes. This work uses a novel integration of machine learning approaches to improve the predicted accuracy of the HHIE-S tool for ARHL in older adults.

**Methods:**

We employed a dataset that was gathered between 2016 and 2018 and included 1,526 senior citizens from several Taipei City Hospital branches. 80% of the data were used for training (*n* = 1220) and 20% were used for testing (*n* = 356). XGBoost, Gradient Boosting, and LightGBM were among the machine learning models that were only used and assessed on the training set. In order to prevent data leakage and overfitting, the Light Gradient Boosting Machine (LGBM) model—which had the greatest AUC of 0.83 (95% CI 0.81–0.85)—was then only used on the holdout testing data.

**Results:**

On the testing set, the LGBM model showed a strong AUC of 0.82 (95% CI 0.79–0.86), far outperforming conventional techniques. Notably, several HHIE-S items and age were found to be significant characteristics. In contrast to traditional HHIE research, which concentrates on the psychological effects of hearing loss, this study combines cutting-edge machine learning techniques—specifically, the LGBM classifier—with the HHIE-S tool. The incorporation of SHAP values enhances the interpretability of the model's predictions and provides a more comprehensive comprehension of the significance of various aspects.

**Conclusions:**

Our methodology highlights the great potential that arises from combining machine learning with validated hearing evaluation instruments such as the HHIE-S. Healthcare practitioners can anticipate ARHL more accurately thanks to this integration, which makes it easier to intervene quickly and precisely.

## Introduction

Age-related hearing loss (ARHL) is a common condition that occurs naturally as individuals age and is characterized by progressive bilateral high-frequency sensorineural hearing loss. As life expectancy increases globally, ARHL has become a significant health concern, particularly for individuals aged 60 and above in both the United States and Taiwan [1, 2]. This age-related condition can lead to various adverse consequences, including difficulties with balance and movement [3–5], social isolation [6], cognitive impairment [6–9], and even an increased risk of mortality [10]. It is crucial to identify and treat ARHL in a timely manner to promote healthy aging and mitigate these negative impacts.

While pure tone audiometry remains the gold standard for detecting hearing loss, its feasibility in large-scale population-based settings may be limited. As a result, there is growing interest in alternative screening techniques that are more accessible and efficient. Recent research has explored various options, such as whisper voice testing [11], telephone-based assessments [12], computer-based evaluations [13, 14], and internet or smartphone applications [15–17], to evaluate hearing sensitivity in older adults. However, questionnaires such as the Traditional Chinese version of the Hearing Handicap Inventory for the Elderly-Screening (TC-HHIE-S) [18] offer a cost-effective and convenient approach to assessing hearing loss, particularly in clinical settings without proper soundproofing. Our previous research has demonstrated that this 10-question questionnaire is a reliable tool for detecting hearing loss and can serve as a suitable alternative to audiometry in large-scale hearing screening initiatives [18].

Declining attention span in older individuals can impact their ability to complete lengthy questionnaires, making it essential to develop a shorter and more efficient screening tool for hearing loss [19]. Additionally, understanding how individual demographics, such as age and gender, influence the accuracy of hearing loss estimation is crucial. Machine learning techniques offer promising solutions to address these challenges. By leveraging data-driven learning without relying on rule-based programming, machine learning algorithms can optimize prediction models using demographic variables and HHIE-S data to achieve accurate hearing loss estimation [20, 21].

The primary objective of this study is to develop a concise version of the HHIE-S questionnaire and evaluate its effectiveness in a Taipei community using a machine learning algorithm [18]. Integrating machine learning into hearing screening protocols can lead to more personalized and effective assessments, improving the overall health outcomes for older individuals with ARHL. It may also help identify high-risk individuals who could benefit from early interventions and support, ultimately contributing to improved quality of life in the aging population.

## Methods

### Data sources and study population

A government-funded annual geriatric health check-up program was available to Taipei residents 65 years of age and older from January 2016 to December 2018. Notified by the city government, eligible participants usually had examinations at neighborhood community hospitals. Participants from Taipei City Hospital's Heping branch, which serves the Zhongzheng and Wanhua districts of Taipei, were the study's primary focus. A total of 1,526 adults (706 men and 820 women, *p* > 0.05) were included in the study.

### Feature selection

At Taipei City Hospital, information was gathered for the training cohort. The participants were given the Hearing Handicap Inventory for the Elderly—Screening (TC-HHIE-S) questionnaire in Traditional Chinese face-to-face, and an audiologist or an undergraduate student studying audiology under supervision recorded the results in their medical records. There were twelve features in the dataset that were analyzed, including demographic variables like gender and age. The TC-HHIE-S questionnaire, comprising ten questions, was also included, offering a thorough understanding of the participants' hearing health.

### Pure-tone audiometry

As the gold standard for detecting hearing loss, pure-tone audiometry was carefully carried out in a sound-treated booth in our study, with ambient noise levels strictly kept below 30 dBA. To perform the audiometry tests, we used the MA30 Audiometer (Maico, Germany) in conjunction with the TDH-39 supra-aural earphones. Four key frequencies—0.5 kHz, 1 kHz, 2 kHz, and 4 kHz—were carefully measured to determine the air conduction pure-tone thresholds. These measurements were primarily taken in the better-hearing ear, with the right ear being used as a default when there was no discernible hearing difference between the two ears. These thresholds were then averaged in the better-hearing ear to determine the pure-tone average (PTA). Narrow band masking was used as needed to guarantee the precision of our measurements, and participants usually indicated their hearing responses with a standard patient response button. An essential component of our approach involved our equipment's yearly calibration, which was conducted with strict adherence to ISO 389–1 and 389–3 guidelines. This step was essential to maintaining the integrity and robustness of our study's findings by ensuring the accuracy and consistency of our audiometric data.

### Class definition

To account for varying degrees of hearing loss, we used a straightforward and methodical method to divide the Pure-Tone Average (PTA) results of study participants into two groups. The established hearing threshold cutoff points served as the basis for this classification. More specifically, individuals with a PTA of less than 40 dB HL—a sign of mild to moderate hearing loss—were given a classification value of 1. In contrast, individuals whose PTA matched or exceeded 40 dB HL were assigned a classification value of 0. The latter category represents hearing loss ranging from severe to profound. We were able to classify the study participants' hearing impairment severity with this binary classification system, which gave us a solid foundation for our machine learning analysis later on.

### Machine learning model development

For model development, we utilized the graphical tool Auto AI in Watson Studio to analyze the 80% training data (*n* = 1220) and identify the best data transformations, algorithms, and parameter settings for our predictive modeling task [22–24]. The Auto AI tool presented the results as candidate model pipelines, which were ranked on a leaderboard, enabling the selection of the optimal model [25–27]. We assessed the performance of six diverse machine learning models on the training data, including extreme gradient boosting (XGBoost) [28–30], gradient boosting classifier (GBC) [31–33], snap decision tree classifier (SDTC) [23, 34, 35], light gradient boosting machine (LGBM) [36–38], snap random forest classifier (SRFC) [26, 27, 39], and logistic regression (LR) [40–42] on the training set, and was thus selected for final evaluation on the 20% held-out testing data (*n* = 306). To improve model efficiency, we implemented forward feature selection on the training data to identify the most informative subset of features. By developing and evaluating models solely on the training set, we prevented leakage and overfitting to the test data.

### Hyperparameter optimization

To optimize the performance of the machine learning models in predicting ARHL, we employed a grid search combined with fivefold cross-validation for XGBoost, GBC, SDTC, LGBM, SRFC, and LR [43–46]. During the training process, we fine-tuned the hyperparameters for each ensemble model using a grid search to identify the optimal values that yielded the highest F1 score [46, 47]. Table 1 presents the detailed hyperparameter optimization settings for each model. By iteratively exploring a predefined set of hyperparameter values through the grid search, we were able to enhance the accuracy and predictive capability of the models in assessing the risk of ARHL. This iterative approach allowed us to find the best combination of hyperparameter values for each model, ultimately leading to improved performance and more reliable predictions.

**Table 1:** Hyperparameters of machine learning models

Model	Hyperparameters	Optimal values
Extreme Gradient Boosting (XGBoost)	learning_rate	0.0384
	max_depth	7
	n_estiators	494
Gradient Boosting Classifier (GBC)	min_samples_leaf	5
	n_estimators	33
Stochastic Dual Coordinate Ascent (SDTC)	max_depth	5
	random_state	33
Light Gradient Boosting Machine (LGBM)	learning_rate	0.415
	n_estiators	566

### Model evaluation

We compared the models' Area Under the Receiver Operating Characteristic Curve (AUC) values to assess the machine learning models' discriminative performance. Because it indicates how well a model can distinguish between classes, the AUC metric is important. We used a suite of performance metrics to conduct a thorough assessment of the models' efficacy on the testing dataset, in addition to AUC. These included the F1 score, which strikes a balance between recall and precision; accuracy, which gauges the model's overall correctness; precision, which shows the percentage of true positive identifications that were correctly identified; recall, which gauges the percentage of true positives that were correctly identified; average precision, which provides a summary of a precision-recall curve; and log loss, which assesses the prediction error of the models. By utilizing this diverse range of metrics, we were able to perform a comprehensive evaluation of the models' efficacy, guaranteeing a detailed and refined comprehension of their potential to forecast age-related hearing loss (ARHL).

### Feature importance analysis

We used SHapley Additive exPlanations (SHAP) analysis to clarify the underlying mechanisms guiding the predictions of our model and to provide guidance for bettering hearing screening procedures. A state-of-the-art approach in explainable AI called SHAP offers a detailed perspective of how each feature influences the model's predictions. Our main objective was to analyze the importance of different elements in the HHIE-S survey. We were able to identify the most important questionnaire items and comprehend their individual effects on the risk prediction of age-related hearing loss (ARHL) by utilizing SHAP values. Through the identification of important ARHL risk factors in our dataset, this analysis was able to offer valuable insights for the development of both our predictive model and the field of hearing health assessment as a whole. It enabled us to see the direction and strength of each feature's influence on the model's output in addition to quantifying each feature's importance.

### Impact on hearing care management

The conclusions drawn from our SHAP analysis have a major impact on how focused interventions for those at risk of hearing loss are developed. Our machine learning model identifies the major factors that influence the risk of age-related hearing loss (ARHL), and this approach opens the door to more customized and efficient hearing care management plans. Comprehending these crucial elements enables medical practitioners to devise interventions that are tailored to each individual's specific requirements and risk profile, while also being responsive to the broad patterns noted in ARHL. This tailored approach is anticipated to substantially improve the effectiveness of interventions, leading to enhanced outcomes in terms of both health and quality of life for older adults afflicted with ARHL. The application of such data-driven, personalized healthcare strategies marks a significant advancement in the field of audiology and geriatric care, promising a more nuanced and impactful approach to managing and mitigating the effects of hearing loss.

### Software and model development

The open-source Scikit-learn library was heavily utilized in conjunction with Python, more especially Python Software Foundation version 3.9, as the main platform for all machine learning analyses in our study. A wide variety of tools and algorithms that were essential for our analysis were provided by this library. To effectively test our models on unseen data, we divided the dataset into training and testing sets at random using the sklearn.model_selection.train_test_split module. We used the XGBoost Python package, which is well-known for its effectiveness and high performance in gradient boosting, to build the XGBoost model. The ensemble of sklearn. Our Gradient Boosting Decision Tree (GBDT) model was developed with the help of the GradientBoostingClassifier module, which constructed an ensemble of weak learners to enable precise prediction-making. The sklearn.ensemble was also used by us. The Extra Trees model, an ensemble approach based on decision trees that provides robustness and feature importance estimation, was built using the ExtraTreesClassifier. The lightgbm. LGBMClassifier Python package, which is preferred for its effectiveness in large-scale applications, was used to develop the Light Gradient Boosting Machine (LGBM) model. A decision tree classifier and sklearn.linear_model were supplied by the SnapDecisionTreeClassifier algorithm from the IBM Snap ML library. The logistic regression model, which is frequently applied to binary classification issues, was utilized. Use sklearn.model_selection to avoid overfitting and guarantee robustness. For stratified k-fold cross-validation, Stratified K Fold was utilized. To ensure compliance with accepted scientific practices, we maintained a p-value of 0.05 to denote statistical significance throughout our analysis.

### Role of the funding source

The study received funding from a specific organization, with the crucial stipulation that the funders remained entirely uninvolved in any aspects of the research process. This encompassed study design, data collection, analysis, interpretation, writing, and paper submission. The clear separation between funding and research activities ensured that the research was conducted independently, without the potential for bias or influence from the funding source. All authors involved in the study had full access to the data, fostering transparency and unbiased research. The corresponding authors assumed final responsibility for paper submission, ensuring that the research adhered to scientific standards and ethical guidelines. They were accountable for upholding the accuracy and integrity of the work presented in the paper.

## Results

### Patient population overview

In this work, we conducted a thorough analysis of a cohort of 1,526 older adults, which is representative of the larger population affected by age-related hearing loss (ARHL). To ensure a thorough evaluation of our machine learning models, this cohort was carefully split into two subsets: a primary training set of 1,221 participants, which was used to develop and refine the models, and a validation testing set of 305 participants, which was used to independently assess the models' performance and predictive accuracy.

### Demographic distribution

The study focused on an elderly population, which is most affected by ARHL, as evidenced by the average age of 74.6 years (± 2.2 years) across the entire cohort in terms of demographic characteristics. Upon closer inspection, we found that the testing set (72.7 years ± 0.6) and the training set (75.2 years ± 2.4) had slightly different average ages. These differences were considered throughout the analysis to guarantee the validity of our results. The cohort's gender distribution was reasonably uniform, with women making up roughly 53.7% of all participants. Table 2 shows that this percentage differed slightly between the training set (53%) and testing set (56.7%). These demographic details are essential because they guarantee that our models are tested on a representative sample of the target population and give context for the findings of our study.

**Table 2:** Demographics and clinical features of Age-Related Hearing Loss (ARHL) patients

Variables	Full Cohort (*n* = 1,526)	Training set (*n* = 1221)	Testing set (*n* = 305)
Demographic			
Age, years	74.1 ± 7.2	74.5 ± 7.4	72.7 ± 6
Female, n (%)	820 (53.7%)	647 (53%)	173 (56.7%)
HHIE-S Item Score			
HHIE-1	0.6 ± 1.2	0.6 ± 1.2	0.2 ± 0.7
HHIE-2	0.5 ± 1.1	0.6 ± 1.2	0.3 ± 0.8
HHIE-3	1.2 ± 1.5	1.3 ± 1.6	0.8 ± 1.3
HHIE-4	0.6 ± 1.3	0.7 ± 1.3	0.3 ± 0.9
HHIE-5	0.4 ± 1	0.5 ± 1.1	0.2 ± 0.6
HHIE-6	0.3 ± 0.9	0.3 ± 1	0.1 ± 0.5
HHIE-7	0.3 ± 0.8	0.3 ± 0.8	0.2 ± 0.6
HHIE-8	0.8 ± 1.4	0.9 ± 1.4	0.5 ± 1
HHIE-9	0.3 ± 0.9	0.4 ± 1	0.1 ± 0.5
HHIE-10	0.6 ± 1.2	0.6 ± 1.2	0.3 ± 0.9

*Abbreviations*: *HHIE-1 *"Do hearing problems embarrass you when you meet new people?", *HHIE-2 *"Does a hearing problem make you feel frustrated when you talk to members of your family?", *HHIE-3 *"Do you find it difficult hearing when someone speaks in a whisper?", *HHIE-4 *"Do you feel that you have a disability because of a hearing problem?", *HHIE-5 *"Does a hearing problem cause you difficulty when visiting friends and relatives or neighbors?", *HHIE-6 *"Does a hearing problem reduce your attendance at religious ceremonies more than you would like?", *HHIE-7 *"Does a hearing problem cause disputes between you and a family member?", *HHIE-8*: "Does a hearing problem cause difficulty when you are listening to the radio or television?" *HHIE-9 *"Do you feel that any difficulty in hearing limits or hinders your personal or social life?”, *HHIE-10 *"Does a hearing problem cause you difficulty when you are in a restaurant with relatives or friends?"

### HHIE-S score insights

A nuanced comparison of HHIE-S scores across the full cohort, training, and testing sets showed subtle differences. For instance, the score for the HHIE-1 item was marginally higher in the full cohort (0.6 ± 1.2) than in the testing set (0.2 ± 0.7), while the training set maintained a score of 0.6 ± 1.2. Similar patterns were observed for the other HHIE-S items, underscoring the consistent representation of hearing impairment symptoms across subsets.

### Model prediction ability

**Fig. 1:**
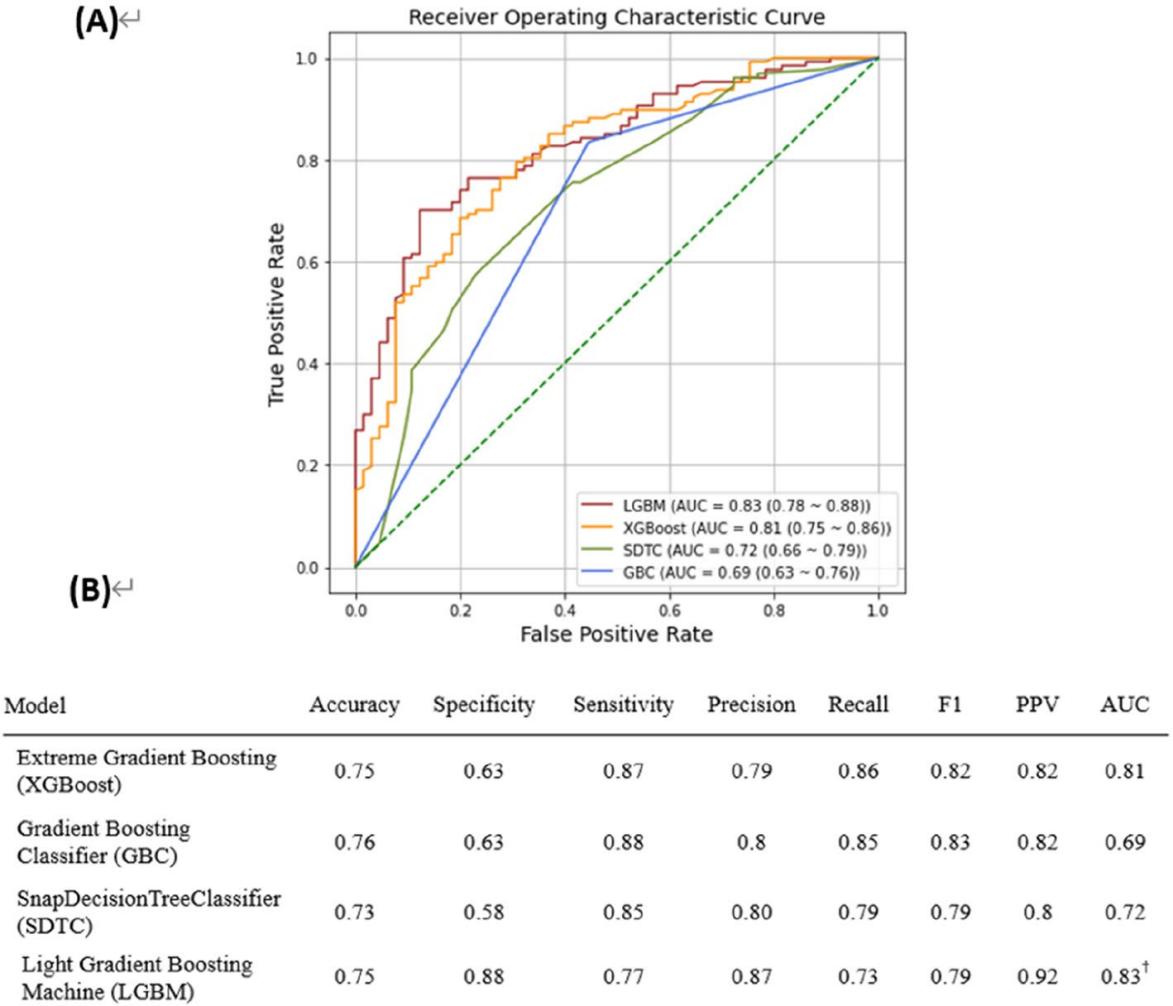
Evaluation of Machine Learning Models for Age-Related Hearing Loss Prediction on the Testing Dataset. **A** Receiver operating characteristic (ROC) curves and **B** LGBM demonstrate the highest area under the ROC curve for age-related hearing loss (ARHL) prediction on the testing dataset, outperforming XGBoost and SDTC. Abbreviations: ROC, receiver operating characteristic; AUC, area under the ROC curve; precision, average precision; extreme gradient boosting (XGBoost), gradient boosting classifier (GBC), snap decision tree classifier (SDTC), light gradient boosting machine (LGBM), snap random forest classifier (SRFC)

Different performance characteristics were observed in our assessment of the predictive capabilities of different machine learning models, such as XGBoost, Gradient Boosting Classifier (GBC), Snap Decision Tree Classifier (SDTC), Light Gradient Boosting Machine (LGBM), and Snap Random Forest Classifier (SRFC). Remarkably, as shown in Fig. 1A, XGBoost and LGBM had the highest accuracy at 0.75, closely followed by GBC at 0.76. In the context of hearing loss predictions, LGBM proved to be a superior model in terms of specificity, achieving an astounding rate of 0.88 in correctly identifying true negative cases. With a score of 0.87, XGBoost showed remarkable sensitivity, demonstrating its ability to accurately identify true positive cases. Precision, recall, and F1 scores were fairly consistent among the models; LGBM and GBC achieved particularly noteworthy scores in these metrics. Furthermore, with the highest positive predictive value (PPV) of 0.92, LGBM stood out. LGBM exhibited the highest Area Under the Curve (AUC) value of 0.83, as illustrated in Fig. 1B, indicating its superior overall discriminating ability. This thorough evaluation provides important insights for the models' use in this field by highlighting the various strengths of each model in predicting age-related hearing loss.

### Feature importance and SHAP analysis in the LGBM model

**Fig. 2:**
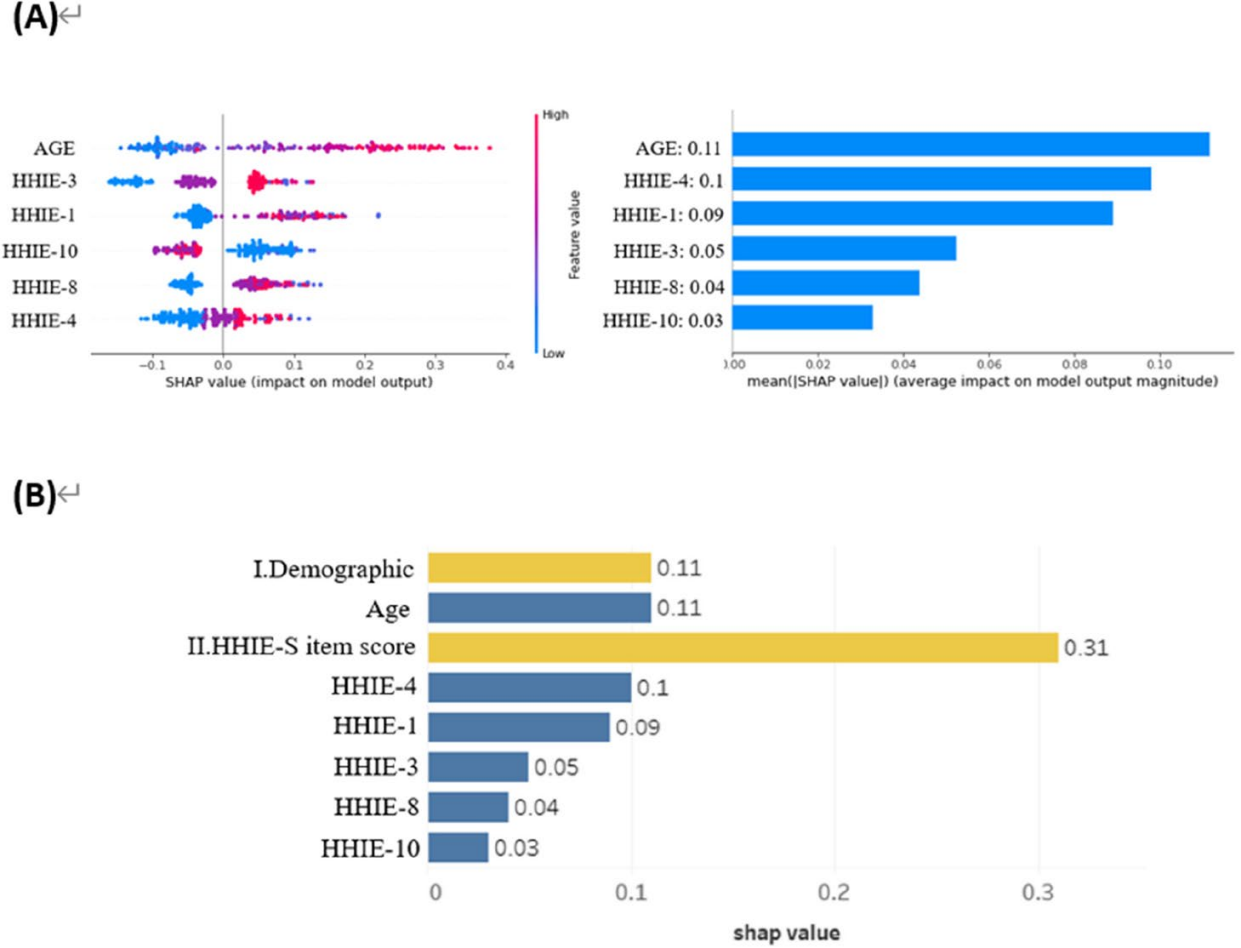
**A** Feature importance plot and **B** SHAP summary plot revealing the top clinical features for predicting age-related hearing loss (ARHL) in the LGBM model. Abbreviations: HHIE-4: "Do you feel that you have a disability because of a hearing problem? ", Age, HHIE-1: "Do hearing problems embarrass you when you meet new people? ", HHIE-3: "Do you find it difficult to hear someone who speaks in a whisper? ", HHIE-8: "Does a hearing problem cause difficulty when you are listening to the radio or television? ", HHIE-10: "Does a hearing problem cause you difficulty when you are in a restaurant with relatives or friends?"

In this work, we employed SHapley Additive exPlanations (SHAP) values to determine the significance of each feature in the Light Gradient Boosting Machine (LGBM) model. As seen in Fig. 2A, this required creating feature importance plots that ordered the most significant features in descending order. AGE, HHIE-4, HHIE-1, HHIE-3, HHIE-8, and HHIE-10 were the most important features influencing the model's predictions, according to our analysis. Higher SHAP values indicated a stronger influence. The SHAP summary plot provided a visual representation of these features' effects on the prediction probabilities of the LGBM model. Significantly, developing Age-Related Hearing Loss (ARHL) was positively correlated with positive SHAP values (red dots), indicating a higher likelihood, and negatively correlated with negative SHAP values (blue dots). As shown in Fig. 2B, our results showed that while HHIE-10 had a negative effect, characteristics like AGE, HHIE-3, HHIE-1, HHIE-8, and HHIE-4 had a positive influence on the prediction of ARHL. This analysis was essential in emphasizing how each of these features individually contributed to the predictive accuracy of the model and in giving users a clear grasp of the critical elements involved in determining an individual's risk of ARHL.

### Individual-level explanation of the ML model

**Fig. 3:**
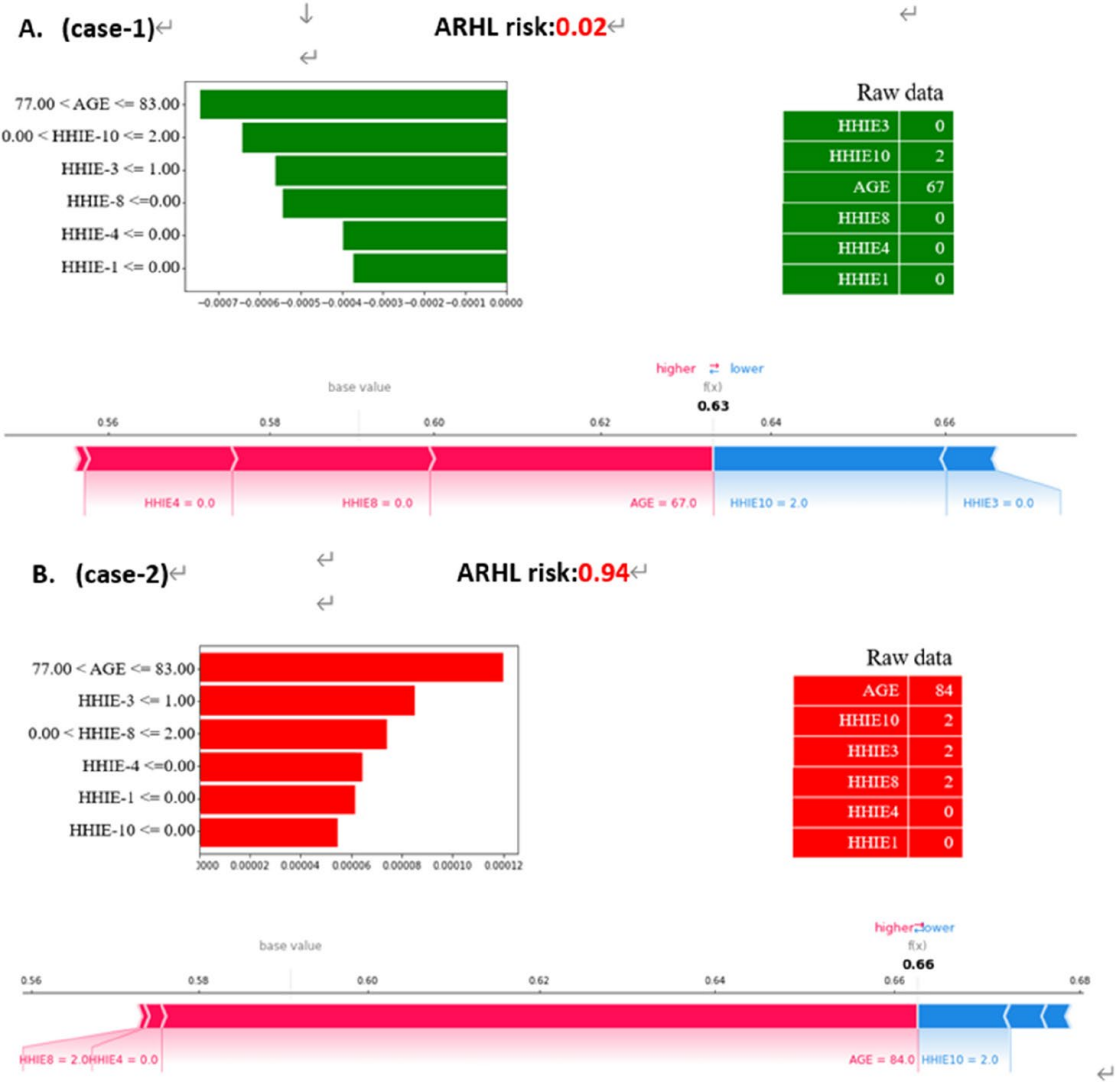
LIME and SHAP Force Plots Visualizing the Impact of Important Features on the Prediction Model for Individual Patients with Age-Related Hearing Loss (ARHL). The plots use color coding, with red indicating a positive correlation and green indicating a negative correlation between the features and the predicted probability of age-related hearing loss (ARHL) risk assessment. Abbreviations: HHIE-4: "Do you feel that you have a disability because of a hearing problem? ", Age, HHIE-1: "Do hearing problems embarrass you when you meet new people? ", HHIE-3: "Do you find it difficult to hear someone who speaks in a whisper? ", HHIE-8: "Does a hearing problem cause difficulty when you are listening to the radio or television? ", HHIE-10: "Does a hearing problem cause you difficulty when you are in a restaurant with relatives or friends?"

For patients at risk of Age-Related Hearing Loss (ARHL), we used SHapley Additive exPlanations (SHAP) and Local Interpretable Model-agnostic Explanations (LIME) to provide comprehensive individual-level explanations of our machine learning (ML) model's predictions. The aforementioned interpretive tools, as illustrated in Fig. 3, efficiently depict the ARHL probability for a pair of representative patients, clarifying the impact of every variable on the probability of hearing impairment. In Case 1, for instance, the LIME plot shows a very low ARHL probability of 0.07, with important contributing factors represented by the red (positive correlation) and blue (negative correlation) variables. These factors include age between 77 and 83, an HHIE-10 score between 0 and 2, and an HHIE-3 score of 1 or less. In contrast, Case 2 shows a 75% chance of ARHL. This higher risk prediction is heavily influenced by similar age parameters combined with scores from HHIE-3, HHIE-8, HHIE-1, and HHIE-10 in particular ranges. These individual-level analyses are essential because they provide a clear and understandable representation of the predictive factors in the model, allowing medical practitioners to obtain in-depth understanding of the model's evaluation of patients' ARHL risk.

## Discussion

In this study, we utilized machine learning techniques to validate the efficacy of the HHIE-S tool in assessing the risk of ARHL [18, 48, 49]. Our results highlight the potential of machine learning models, especially LGBM, to enhance the predictive capabilities of the HHIE-S tool by incorporating demographic and clinical features [50–52]. Our research is in line with the increasing interest in leveraging machine learning in audiology and hearing healthcare, as evident from recent publications [53–55]. Numerous studies have been dedicated to the development and validation of machine learning models for predicting hearing loss, evaluating interventions, and improving diagnostic accuracy [56–58].

In our study, the LGBM model demonstrated the best specificity (0.88), showcasing its proficiency in accurately identifying true negative cases. Additionally, it exhibited the highest PPV of 0.92, indicating its capability to correctly predict positive cases with high confidence. The AUC analysis revealed that the LGBM model achieved the highest AUC of 0.83, signifying its superior overall discriminative ability. These results align with our previous findings, where PTA > 40 dB HL showed the highest sensitivity (76.9%) in hearing screening, indicating similar discriminatory power [18].

The HHIE-S tool is a cost-effective and easily applicable assessment, making it suitable for diverse settings such as nursing homes, senior centers, primary care facilities, and community hearing screenings with numerous participants within a short timeframe. These locations often lack access to conventional hearing tests. This research emphasizes the significance of the HHIE-S in public health applications, as it can identify moderate and severe hearing loss in a considerable population. To further enhance the practicability and effectiveness of large-scale HHIE-S screenings, future studies should investigate the utilization of internet-based or smartphone-based methods for managing HHIE-S [59, 60].

By employing LGBM models in our study, we observed enhanced predictive performance compared to that of traditional statistical methods. Notably, the LGBM achieved the highest AUC values, indicating its superior ability to predict ARHL risk. This result aligns with recent findings of the effectiveness of LGBM and other ensemble models in various medical applications. Conducting a feature importance analysis using SHAP values provided valuable insights into the most influential features [61]. Age and specific HHIE-S questionnaire items, particularly HHIE-4, emerged as the top predictors [18, 48, 62]. These results are consistent with previous findings that have emphasized the role of age and self-perceived hearing difficulties in predicting hearing loss.

Six features on the importance matrix plot and the SHAP summary plot of LGBM were age and the five HHIE-S results. The most important factor for the model output was "Do you feel that you have a disability because of a hearing problem?": Our model identified the 4th item of the HHIE-S questionnaire as the most influential in determining hearing loss. This question directly addresses the issue of hearing loss in a clear and understandable way. It measures emotional responses to hearing loss and is similar to the commonly used single question (SQ): "Do you have any difficulty with your hearing?", which has shown reasonable effectiveness in detecting hearing loss during large-scale screenings [63–65].

### Age

Age is an important variable in detecting hearing loss in our model. Our study aligns with several studies that demonstrated age as a significant variable in questionnaires for detecting hearing loss [66]. The significance may be due, in part, to the fact that the prevalence of ARHL increases with age [67–69]. Although some studies also indicate that older age could lead to overestimation or underestimation in self-reported hearing loss compared to objective hearing loss, it may be attributed to the social acceptability of older individuals experiencing hearing impairment [70–73]. Nonetheless, including age as a variable in a hearing screening session is convenient because age data are easily accessible in any massive screening situation [68, 72, 74, 75].

### Do you find it difficult to hear someone who speaks in a whisper?

Our analysis shows that the whisper test is the second most significant factor in measuring hearing loss. This is an interesting finding, as the whisper test is a simple and accurate screening method widely used in hearing screenings for elderly individuals, with reasonable sensitivity and specificity [64, 76–78]. In fact, the World Health Organization recommends using the whisper test in hearing screenings as part of the Integrated Care for Older People (ICOPE) program [79]. During a whisper test, air is pushed through a small opening between the vocal cords without adduction, resulting in a quieter, breathier sound that may be less clear and distinct, making it more difficult to understand the speaker, especially in noisy environments or at a distance. Whispering is typically used to convey secret information or avoid disturbing others in a quiet setting, and failure to hear whispered sounds can cause individuals to perceive hearing loss in these situations.

### Does a hearing problem cause difficulty when you are listening to the radio or television?

TV and radio are currently very common forms of media. However, a study has suggested that patients who report increased volume levels while watching TV may have an increased risk of hearing loss [80]. It is possible that individuals who have difficulty hearing the sounds from these devices may assume they have hearing loss when, in fact, they may simply be unable to detect certain frequencies or volumes of sound. Further research is needed to clarify the relationship between increased TV volume and hearing loss.

### Does a hearing problem cause you difficulty when you are in a restaurant with relatives or friends?

Restaurants are bustling environments filled with sounds such as background music, conversations, and the clanging of utensils, which can make it challenging for people with hearing loss to discern between sounds or comprehend speech. In fact, speech understanding difficulties in noisy environments are frequently cited as the top reason for consultations in audiology or hearing health services [81]. It is important to conduct further research to better understand this association.

Our study adds to the expanding body of evidence supporting the integration of machine learning models into hearing screening protocols. By accurately identifying individuals at risk of ARHL, healthcare professionals can implement timely interventions, such as hearing aids or rehabilitation programs, to mitigate the impact of hearing loss on quality of life. The use of machine learning models enhances the efficiency and accuracy of screening processes, leading to improved patient outcomes. However, it is essential to acknowledge several limitations in our study. Focusing on a specific population of older adults limits the generalizability of our findings to other age groups or populations [62]. Future research should aim to validate the HHIE-S tool and machine learning models in diverse populations to ensure their applicability across settings. Additionally, the quality and availability of data may influence model performance. Future studies should consider larger sample sizes and more comprehensive datasets to enhance the robustness and generalizability of the models.

### Comparison with traditional HHIE approaches

Our study significantly extends the utility of the Hearing Handicap Inventory for the Elderly (HHIE) by integrating it with cutting-edge machine learning models. Traditionally, HHIE has been used primarily to assess the psychosocial impacts of hearing impairment through subjective experiences and self-perceived hearing difficulties. This novel method improves the precision and accuracy of Age-Related Hearing Loss (ARHL) predictions and introduces a more individualized approach to hearing healthcare by transforming HHIE from a simple self-reporting tool into an advanced predictive tool. We can customize interventions based on each patient's unique risk profile by utilizing machine learning, which combines quantitative, data-driven predictions with qualitative insights into patients' actual experiences. This combination marks a significant breakthrough in the field of hearing healthcare and the start of a new phase of proactive, patient-centered, precision-based care. Our approach provides a comprehensive and nuanced view of the management of hearing health by bridging the gap between subjective self-assessment and objective risk prediction. This adds quantitative and data-driven capabilities to the traditional HHIE approach, enabling a more holistic approach to hearing healthcare.

## Conclusion

Our research, which combines cutting-edge machine learning methods with the traditional Hearing Handicap Inventory for the Elderly-Screening (HHIE-S) tool, marks a major advancement in the assessment of hearing loss. In the field of audiology and hearing healthcare, the use of the Light Gradient Boosting Machine (LGBM) classifier in particular has shown impressive progress in improving predictive accuracy. In addition to demonstrating the enormous potential of data-driven approaches to supplement conventional diagnostic tools, this study paves the way for more prompt and precisely targeted interventions for age-related hearing loss (ARHL). The significance of adopting such novel approaches is highlighted by our study, as the healthcare sector continues to undergo swift transformation. Ensuring better patient outcomes and leading the way in the transition to a more proactive, precision-driven paradigm in hearing care depend on this. By utilizing machine learning, we create new opportunities for early detection and intervention, which will ultimately improve the quality of life for those who are impacted by hearing loss.

## Data Availability

The data used in this study are not publicly available, as they were obtained from Taipei City Hospital for Department of Otorhinolaryngology, under a license agreement. The data belong to Tzong-Hann Yang, who is one of the authors of this study. If you have a reasonable request for accessing the data, you can contact Tzong-Hann Yang and ask for his permission. He will decide whether to grant you access to the data, based on the terms and conditions of the license agreement with Taipei City Hospital. Potential conflicts of interest to declare.
